# Estimating a minimal clinically important difference for the EuroQol 5-dimension health status index in persons with multiple sclerosis

**DOI:** 10.1186/1477-7525-12-66

**Published:** 2014-05-05

**Authors:** Christine G Kohn, Matthew F Sidovar, Kirandeep Kaur, Yungfen Zhu, Craig I Coleman

**Affiliations:** 1 Department of Pharmacy Practice, University of Connecticut School of Pharmacy, Storrs, CT, USA; 2 Hartford Hospital Evidence-Based Practice Center, Hartford, CT, USA; 3 Clinical Development and Medical Affairs, Acorda Therapeutics, Ardsley, NY, USA

**Keywords:** Multiple sclerosis, Quality of life, Health status, EuroQol 5-Dimension, Minimal clinically important difference

## Abstract

**Background:**

Limited data define what constitutes a minimal clinically important difference (MCID) on the EuroQol 5-Dimension (EQ-5D) health status index in persons with multiple sclerosis (PwMS). We sought to estimate the MCID for the EQ-5D health index in North American PwMS.

**Methods:**

PwMS completing the Patient Determined Disease Steps (PDDS) scale, 12-Item Multiple Sclerosis Walking Scale (MSWS-12) and EQ-5D as part of the North American Research Committee on Multiple Sclerosis (NARCOMS) registry’s spring 2011 update and supplemental survey were included in this retrospective, cross-sectional study. Distribution-based (standard error of measurement [SEM], 0.50 standard deviation [SD] and 0.33 SD unit) approaches were used to estimate a range of MCIDs for the EQ-5D based upon disease severity groups determined by the PDDS and MSWS-12 tertiles.

**Results:**

A total of 3,044 participants were included. Moderately strong correlations between the EQ-5D and the PDDS and MSWS-12 were observed (Spearman’s r = -0.56 and -0.59, respectively, p < 0.0001 for both). MCID estimates based on PDDS score categories ranged from 0.065-0.158 (SEMs), 0.059-0.142 (0.50 SDs) and 0.039-0.095 (0.33 SDs). MCID estimates as measured by MSWS-12 tertile categories ranged from 0.068-0.098 (SEMs), 0.061-0.088 (0.50 SDs), and 0.041-0.059 (0.33 SDs). Across both the PDDS and tertiles of MSWS-12, MCID estimates tended to be larger as disease severity worsened. Mean weighted MCID estimates ranged from 0.05-0.084 for both the PDDS and MSWS-12 tertiles.

**Conclusion:**

MCID estimates for the EQ-5D in PwMS were within the range of estimates seen for other disease states and appeared to be larger in those reporting more severe disease.

## Introduction

Multiple sclerosis (MS) has long been known to negatively impact health-related quality of life (HRQoL) [1-3]. For this reason, clinical studies have increasingly utilized validated HRQoL measures to evaluate the effect of treatment or the impact of disease progression on HRQoL in persons with MS (PwMS) [4-6]. The Euro-Qol 5-Dimension (EQ-5D), a generic health status index score [7,8], is one such measure; however, interpretation of the EQ-5D in PwMS is hampered by a paucity of information regarding what constitutes a minimally important clinical improvement in score in this population. The purpose of this study was to estimate the minimal clinically important difference (MCID), or smallest difference in score PwMS from North America perceive as being both beneficial and nontrivial [9].

## Methods

We used data from the North American Research Committee on Multiple Sclerosis (NARCOMS) registry to conduct this retrospective, cross-sectional analysis [10]. NARCOMS gathers self-reported patient data through an extensive, semi-annual health survey of registrants. In 2010, NARCOMS, in conjunction with Acorda Therapeutics, Inc., sent a supplemental questionnaire to a sample of 4,389 NARCOMS registrants reporting a Patient-Determined Disease Step (PDDS) score ≤7 to collect additional data on patient perceptions of HRQoL using the EQ-5D, 3-level (3 L) descriptive system and walking using the 12-item Multiple Sclerosis Walking Scale (MSWS-12). The supplemental survey was sent about one month after the close of the update survey.

The collection and research use of NARCOMS data is approved by the Institutional Review Board (IRB) at the University of Alabama at Birmingham. Approval was obtained from the same IRB for the acquisition of the additional data via the supplemental questionnaire. The secondary analyses reported here were reviewed and approved by the IRB at Hartford Hospital and conducted with de-identified datasets.

PwMS who completed the EQ-5D, PDDS and MSWS-12 as part of the NARCOMS update and supplemental surveys during spring 2010 were included in this study. The EQ-5D-3 L consists of five descriptive questions concerning five domains of HRQoL (mobility, self-care, usual activities, pain/discomfort, anxiety/depression) each with 3-levels of response (no, some/moderate or unable/extreme) with participants’ pattern of responses used to derive health status index scores. US-specific health status index scores between 1.0 and -0.11 (on a scale where 1.0 = perfect health and 0.0 = death, negative values suggest health states worse than death) were derived using the scoring algorithm developed in a study of a general US population [7].

Disease severity in PwMS was evaluated using the PDDS, as the Kurtzke Expanded Disability Status Scale (EDSS) and other clinician-based tools (signs, symptoms and medical examination results) are not available in the NARCOMS registry. The PDDS is a patient-reported measure that is scored ordinally from 0 (no disability) to 8 (bedbound) [11]. It has been shown to correlate well with the neurologist scored EDSS (r = 0.78; p = 0.0001) [12]; and both measures have been used by clinicians and researchers to assess MS-related disease severity and progression.

PwMS also completed the MSWS-12, a validated, patient-reported outcome measure of walking impairment that consists of 12 questions with five possible responses ranging from 1 (not at all) to 5 (extremely) [13]. Each question represents a different aspect of walking function and quality, including walking speed, ability to run, ability to climb and descend stairs, ability to stand, balance, endurance, smoothness of gait, need for support (indoors and outdoors), and concentration required. The possible scores for this measure range from 12–60, and were converted to scores ranging to 0–100 for easier interpretation, with higher scores representing greater walking disability [13]. The MSWS-12 is the only measure of walking ability reported in the NARCOMS registry and is strongly correlated with both the PDDS (r = 0.80; p < 0.01) and EDSS (r = 0.73; p < 0.01) (since both the PDDS and EDSS focus on mobility in the middle of their scales) [12].

Since no suitable anchor was available in our NARCOMS dataset, multiple distribution-based approaches were used to estimate MCIDs for the EQ-5D in this NARCOMS cohort. Consistent with prior studies, we used the following distribution-based approaches: (1) the standard error of measurement (SEM), (2) 0.5 * standard deviation (SD) and (3) 0.33 * SD [9,14-19]. The SEM describes the variability between an individual’s observed score and the true score and is calculated as the SD of the measure multiplied by the square root of 1 minus its reliability coefficient [9,14]. For this analysis, we used Cronbach’s alpha as the measure of reliability of the EQ-5D when calculating the SEM. Our estimate of Cronbach’s alpha in this NARCOMS population was 0.69, which is consistent with estimates reported in prior studies [19].

Correlations between the PDDS, MSWS-12 and the EQ-5D were assessed using Spearman’s rho (r), with a coefficient ≥0.40 suggesting an adequate correlation. We used the PDDS to define disease severity in PwMS (using the ordinal values from 0–8) and the MSWS-12 tertiles (with scores of 0 – 33 representing mild walking impairment; 34 – 67 as moderate walking impairment; and 68 – 100 representing severe walking impairment) to grouppatients into walking impairment categories. Subsequently, we applied the above-mentioned distribution-based approaches to each category. Resulting MCID estimates were summarized as ranges, and a mean MCID across categories, weighted by sample size, was calculated for each questionnaire.

Descriptive statistics including percentages for categorical data; and medians/ranges and means/SDs for ordinal/continuous data are reported. All data analyses for this study were conducted with SPSS version 20.0 (SPSS Inc., Chicago, IL).

## Results

In 2010, 3,609 subjects completed the spring NARCOMS update and supplemental surveys. Of those, 3,307 subjects also completed the PDDS, 3,338 completed MSWS-12 and 3,557 completed the EQ-5D. Therefore, of the 4,389 originally sent both surveys, a total of 3,044 NARCOMS registrants (69.4%) had complete data sets and were included in this analysis. Characteristics of these PwMS are depicted in Table 1. Most PwMS were women, Caucasian, and on average, in their fifth decade of life. Mean duration in time from initial MS diagnosis was nearly 18 years, and 61.4% had received a disease-modifying drug within the prior 6 months. Approximately 63% were not working or attending school. The mean transformed MSWS-12 score for the entire population was 50.2 ± 33.7 (median and range = 52, 0–100), with PwMS relatively evenly distributed among the three walking impairment tertiles. The median PDDS of the population was 3, with at least three-quarters having a score of 5 or below. EQ-5D index scores ranged from a minimum of -0.04 to a maximum of 1.0 (0% and 13.3% at the floor and ceiling, respectively) with a mean score of 0.74 ± 0.18 and a median score (range) of 0.78 (-0.04-1.0). Moderately-strong correlations between the EQ-5D and the PDDS and MSWS-12 were observed (Spearman’s r = -0.56 and -0.59, respectively, p < 0.0001 for both).

**Table 1:** Characteristics of study population

**Characteristics**	**Patients (n = 3,044)**
Age (mean, SD)	56.8 (9.9)
Female (n, %)	2444 (80.3%)
Race	
White	2875 (94.4%)
Non-White	127 (4.2%)
Unknown	42 (1.4%)
Duration (avg. years, SD)	17.9 (8.9)
DMD in previous 6 months (n, %)	1869 (61.4)*
Currently working/attending school (n, %)	1121 (36.8)
PDDS Score (Median, IQR)	3 (1, 5)
0	526 (17.3%)
1	428 (14.1%)
2	241 (7.9%)
3	429 (14.0%)
4	507 (16.7%)
5	427 (14.0%)
6	399 (13.1%)
7	83 (2.7%)
8	4 (0.13%)
MSWS-12 Score (Mean, SD)	50.2 (33.7)
Median (Range)	52 (0–100)
Tertile 1	1138 (37.4%)
Tertile 2	763 (25.1%)
Tertile 3	1143 (37.5%)
EQ-5D (Mean, SD)	0.74 (0.18)
Median (Range)	0.78 (-0.04-1.0)
Floor (n, %)	0 (0%)
Ceiling (n, %)	404 (13.3%)

n = number of patients; PDDS = Patient Determined Disease Steps; MSWS-12 = 12-point Multiple Sclerosis Walking Scale; EQ-5D = EuroQol-5 Dimension Health Status Index; SD = Standard Deviation; avg = average; IQR = interquartile range; DMD = Disease-Modifying Drug; *Patients may have received ≥1 DMD in previous 6 months.

MCID estimates for EQ-5D index score within PDDS score categories ranged from 0.065-0.158 (SEMs), 0.059-0.142 (0.50 SDs) and 0.039-0.095 (0.33 SDs) (Table 2). MCID estimates within MSWS-12 tertile categories ranged from 0.068-0.098 (SEMs), 0.061-0.088 (0.50 SDs), and 0.041-0.059 (0.33 SDs) (Table 3). Across both the PDDS and tertiles of MSWS-12, MCID estimates tended to get larger as disease severity worsened. Mean weighted MCID estimates ranged from 0.050-0.084 for both the PDDS and MSWS-12 tertiles.

**Table 2:** MCID estimates for EQ-5D based on PDDS subgroups

**EQ-5D scores**									
PDDS*	**N**	**Mean**	**SD**	**Median**	**Min**	**Max**	**SEM**	**0.5 SD**	**0.33 SD**
**0**	526	0.900	0.119	1.000	0.26	1.00	0.066	0.060	0.040
**1**	428	0.834	0.117	0.827	0.37	1.00	0.065	0.059	0.039
**2**	241	0.734	0.147	0.778	0.29	1.00	0.082	0.074	0.049
**3**	429	0.730	0.163	0.778	0.20	1.00	0.091	0.081	0.054
**4**	507	0.689	0.167	0.761	0.17	1.00	0.093	0.084	0.056
**5**	427	0.662	0.171	0.708	0.05	1.00	0.095	0.086	0.057
**6**	399	0.649	0.177	0.705	0.05	1.00	0.099	0.089	0.059
**7**	83	0.616	0.182	0.689	-0.04	0.85	0.101	0.091	0.061
**8**	4	0.463	0.284	0.410	0.18	0.85	0.158	0.142	0.095
**Mean Weighted MCID**	3044	---	---	---	---	---	0.084	0.076	0.051

N = number of patients; PDDS = Patient Determined Disease Steps; SEM = standard error of the measurement; SD = standard deviation.

*PDDS Score 0 = mild to no symptoms; 1 = mild disability; 2 = moderate disability; 3 = gait disability; 4 = early cane; 5 = late cane; 6 = bilateral support; 7 = wheel chair/scooter; 8 = bedridden.

**Table 3:** MCID estimates for EQ-5D based on MSWS12 tertile subgroups

**EQ-5D scores**									
**MSWS12 tertiles**	**N**	**Mean**	**SD**	**Median**	**Min**	**Max**	**SEM**	**0.5 SD**	**0.33 SD**
**1 (0–33)**	1138	0.861	0.123	0.833	0.29	1.00	0.068	0.061	0.041
**2 (34–67)**	763	0.718	0.154	0.778	0.20	1.00	0.086	0.077	0.051
**3 (68–100)**	1143	0.644	0.176	0.708	-0.04	0.85	0.098	0.088	0.059
**Mean Weighted MCID**	3044	---	---	---	---	---	0.084	0.075	0.050

N = number of patients; MSWS12 Tertiles = Multiple Sclerosis Walking Scale coded into tertiles; SEM = standard error of the measurement; SD = standard deviation.

## Discussion

HRQoL assessments can be used by researchers in clinical trials and clinicians and patients in real-world practice settings to measure response to treatment and quantify the impact of changes in disease severity; however, understanding what constitutes a minimally important change on a HRQoL measure is critical for doing so. This is the first study to calculate MCID estimates for the US-scored EQ-5D health status index in PwMS. Our analysis suggests the MCID for a population with MS likely falls between the values of 0.050-0.084. However, our analysis also suggests that the MCID varies by disease severity; with higher estimates in those reporting greater disability due to MS.

Prior studies have estimated MCID values for the US-algorithm scored EQ-5D index in a variety of disease states other than MS [17-20]. Luo and colleagues estimated EQ-5D index scores in patients suffering leg ulcers, early rheumatoid arthritis, back pain, and systemic sclerosis, among others, using distribution-based approaches [17]. They estimated mean MCID estimates that were on the lower end of our calculated ranges (0.040 for US-based EQ-5D). In a study of intervertebral disk herniation, anchor-based MCID estimates for the US-scored EQ-5D ranged from 0.08-0.17 depending on the anchor used [20]. Le and colleagues found MCID estimates ranging between 0.05 and 0.08 when using anchor-based approaches and 0.04-0.10 using distribution-based approaches in a cohort of patients suffering from post-traumatic stress disorder [18]. Yet, another study conducted by Pickard and colleagues using a similar methodology to our own suggested MCID estimates for US-based EQ-5D scores ranged from 0.07-0.09 in patients with cancer, and between 0.04 and 0.07 across various types of cancer [19]. While our range of MCID estimates for the EQ-5D, 0.050-0.084, fall within these previously reported ranges; these prior studies suggest that a single MCID estimate for the EQ-5D does not fit all.

A number of additional points should be considered when interpreting our findings. First, given the cross-sectional nature of the study and the exclusive use of distribution-based methods, our MCID estimates are essentially “between-patient” differences, rather than “within-patient” changes over time. Therefore, our estimated MCIDs are best suited for discriminating between patient groups, as compared to defining responders to treatment. Second, the population in the NARCOMS registry used for this study may not be representative of all MS patients, particularly those living outside of North America and those with PDDS scores of 8. Moreover, NARCOMS data comes from semi-annual surveys, and therefore, responses may be subject to reporting or recall bias. However, despite these limitations stemming from the registry’s design, it provides a unique ability to look at a large (n > 3,000) population of PwMS with varying degrees of disease severity (PDDS range: 0–8; EQ-5D range: -0.04-1.0). This is important because MCID estimates for the EQ-5D index score may vary to an important degree between persons in the same target cohort [18,19]. In our analysis, the MCID was found to be largest in PwMS having the most severe disease or walking impairment, suggesting that patients perceive HRQoL differently based the severity of their current health state. We recommend using the MCID estimate that best matches the population being studied/evaluated (i.e., a MCID of 0.08-0.09 may be more appropriate for patients with greater mobility impairment; while a estimate of ~0.06 may be more suitable for those less impaired). Third, in order to provide a range of plausible EQ-5D MCIDs for PwMS based on disease severity, we used the PDDS and MSWS-12 to define disease severity categories. While we used the native ordinal scale to categorize PwMS using the PDDS, it should be noted we arbitrarily divided the MSWS-12 into equal tertiles based upon score (with scores of 0 – 33 representing mild walking impairment; 34 – 67 as moderate walking impairment; and 68 – 100 representing severe walking impairment). Next, our analysis did not utilize an anchor-based approach, which is often considered more desirable than the distribution-based approaches we used for MCID estimation [9,14]. Unfortunately, the lack of a true anchoring question (one that allows for categorization of patients who perceived only minimal improvement) in the NARCOMS registry precluded the use of an anchor-based approach [9]. A common concern of distribution-based approaches is that they assume data is normally distributed, and estimates can be skewed when large floor or ceiling effects are present [9,14]. However, this was less of a concern in our analysis, since no PwMS were at the floor (lowest possible) EQ-5D value (0% at -0.11) and only a small number were at the ceiling (highest possible) EQ-5D score (13.3% at 1.00). A final consideration for our analysis relates to its external validity. Our use of a North American population and the US population-based EQ-5D scoring algorithm likely means our results are not highly externally valid to PwMS outside the US. In fact, studies suggest EQ-5D index values and MCID estimates using the US scoring algorithm vary significantly from non-US population values because of differences in the scaling properties of the EQ-5D from country to country, as well as potential variation in population characteristics [7,8,21]. For example, Johnson and colleagues [21] compared valuations of the same 42 EQ-5D health states for US and UK populations, and found US mean scores to be numerically higher than the UK for 39 of the health states, with an average increase in score of 0.10 (p < 0.001) for US-based estimates compared to UK estimates after adjusting for known predictors.

## Conclusions

In conclusion, the MCID estimate calculated in this study can aid researchers and clinicians when discriminating between patient groups for EQ-5D index scores of PwMS. Our MCID range of 0.050-0.084 for EQ-5D was within the range of MCID estimates of other disease states. In general, patients who have severe disability had higher MCIDs than patients who had mild-moderate disability. Additional analysis to verify these EQ-5D health status index MCID estimates in an independent data set should be performed.

## Abbreviations

EDSS: Expanded Disability Status Scale; EQ-5D: EuroQol 5-Dimension; MS: Multiple sclerosis; MSWS-12: 12-Item Multiple Sclerosis Walking Scale; NARCOMS: North American Research Committee on Multiple Sclerosis; PDDS: Patient Determined Disease Steps; PwMS: Persons with multiple sclerosis; SEM: Standard error of measurement; SD: Standard deviation.

## Competing interests

This research was funded by Acorda Therapeutics, Inc., Ardsely, NY. The NARCOMS registry is supported in part by the Consortium of Multiple Sclerosis Centers and its Foundation. The publication of these study results was not contingent on the sponsor’s approval or censorship of the manuscript. Mr. Sidovar is a paid employee and stockholder of Acorda Therapeutics, Inc., Ardsley, NY; Dr. Coleman has received research funding from Acorda Therapeutics, Inc., Ardsley, NY; Drs. Kohn, Kaur, Zhu have no conflicts of interest to report.

## Authors’ contributions

Drs CGK and CIC had full access to all of the data in the study and take responsibility for the integrity of the data and the accuracy of the data analysis. Study concept and design: CGK, MFS, CIC. Acquisition of data: CGK, MFS, KK, YZ, CIC. Analysis and interpretation of data: CGK, MFS, KK, YZ, CIC. Drafting of the manuscript: CGK, MFS, KK, YZ, CIC. Critical revision of the manuscript for important intellectual content: CGK, MFS, CIC. Administrative, technical, or material support: CGK and CIC. Study supervision: CIC. All authors read and approved the final manuscript.
